# Functional Analysis of Lactic Acid Bacteria and Bifidobacteria and Their Effects on Human Health

**DOI:** 10.3390/foods11152293

**Published:** 2022-08-01

**Authors:** María Teresa Dueñas, Paloma López

**Affiliations:** 1 Faculty of Chemistry, University of the Basque Country (UPV/EHU), 20018 San Sebastián, Spain; mariateresa.duenas@ehu.eus; 2 Centro de Investigaciones Biológicas Margarita Salas (CIB, CSIC), 28040 Madrid, Spain

Many lactic acid bacteria (LAB) and Bifidobacteria are beneficial components of human, animal, foods, and beverage microbiota. Due to their probiotic properties, and the functionality of the metabolites that they produce, they are widely used as components, starters, and adjunct cultures in dairy products and beverages. Moreover, their use is extending to other sectors such as bakery and meat products.

The probiotic properties of bacteria as well as the metabolites produced by them are strain specific. Therefore, in this Special Issue, LAB and bifidobacteria with potential beneficial influence against human viral infections [1,2] or obesity [3] were investigated. Moreover, the current knowledge and future perspectives concerning the role of postbiotics isolated from LAB in diabetes mellitus is presented [4]. Thus, the effect of *Ligilactobacillus salivarius* MP101 administrated in a dairy product to elderly people in a nursing home was evaluated. The functional (Barthel index) and the nutritional (MNA score) values of the studied population improved significantly after the trial. Furthermore, the concentrations of several inflammation-related immune factors were altered after the trial, with the results indicating that *L. salivarius* MP101 could be a potential probiotic for elderly people [1]. In addition, the antiviral and immunological influence of *Lactobacillus mucosae* 1025 and *Bifidobacterium breve* CCFM1026 were investigated in a mouse model of influenza virus infection. The results showed that a combination of both bacteria reduces the mouse mortality and indicated that the antiviral mechanism is due to inhibition of the viral loading because of increased expression of the antiviral protein MxA, which was closely associated with increased butyrate production due to gut microbiota alteration [2]. Furthermore, Thirty-one LAB, isolated from kimchi in South Korea, were analyzed in vitro for anti-obesity properties, and among them, four were selected for testing in an obese C57BL/6J mouse model. Reduction of obesity was evaluated by weight measurement and serum analyses. The expression of liver genes involved in obesity, and the levels of antioxidant proteins in the liver were also investigated. The results supported that *L. fermentum* SMFM2017-NK4 has anti-obesity effects by inhibiting fat accumulation [3]. Postbiotics consist of bacterial cell structures, their secreted molecules or metabolic by-products, and/or dead microorganisms. The review mentioned above [4] describes the effects on diabetes mellitus of exopolysaccharides, gamma-aminobutyric acid (GABA), extracellular vesicles, supernatants, extracts, surfactants, and dead microorganisms. The authors concluded that postbiotics have potential as new therapeutic agents to combat diabetes mellitus [4].

The metabolic products from LAB such as some exopolysaccharides (EPS) and vitamins have other beneficial properties for human health, such as anti-inflammatory and immunomodulatory activities. Moreover, their production in situ during food matrix fermentation can generate functional food. Thus, in this Special Issue, forty-two LAB were isolated from fermented doughs, and twenty-one of them were characterized as homopolysaccharide (dextran) and vitamin B_2_ producers, with the aim of their future usage for preparation of multifunctional bread [5]. Moreover, the biological functions of LAB exopolysaccharides were reviewed in this Special Issue and the potential benefits for human and farmed animals were detailed and discussed [6].

The EPS play a role in the organoleptic properties of food and beverages. In the first case, their effect is positive. However, for alcoholic beverages the influence of the EPS is controversial, since traditionally the presence of β-glucan synthesized by LAB generate the “ropy” phenotype that provoke the spoilage of cider and wine. Thus, in this Special Issue, the current knowledge of EPS production by LAB in alcoholic beverages (wine, cider, etc.), was reviewed, including their beverage spoilage characteristics and their influence on wine sensorial properties [7]. Additionally, LAB produce EPS during fermentation of Kefir grains. In addition, in this Special Issue includes a review describing the characteristics and identification of probiotics from *Lacticaseibacillus paracasei*. A special emphasis on description and functional properties of the EPS produced by these LAB has been made [8].

Additionally, LAB isolated from Mexican kefir grains and belonging to *Lactococcus*, *Lactobacillus*, and *Leuconostoc* genera were characterized. Their probiotic characteristics, including, among others, resistance to the gastrointestinal stress, adhesion to enterocytes, permeability to prebiotics, and GABA production were investigated with the future aim of utilization of these bacteria for elaboration of functional dairy products [9].

Furthermore, another food matrix (camel milk) was used for the isolation and identification of six GABA-producing *Lactococcus lactis* strains. The technological properties of these LAB were investigated as well as their performance during experimental cabrales-like mini cheese making [10].

In addition, one hundred and six LAB were isolated from fermented meat (pancetta and prosciutto). The molecular identification (determination of the 16S rRNA coding gene sequence) of the strains revealed that the most predominant species was *Lactiplantibacillus plantarum*. Analysis of their potential probiotic properties revealed that *L. plantarum* 41G isolated from prosciutto was the best candidate and that fermented meat is a good substrate to isolate probiotic LAB [11].

Finally, food preservation and safety is another issue where LAB can play a positive role. Therefore, in this Special Issue, a work is included in which the effect of the nisin A-producing *Lactococcus lactis* ssp. *lactis* 32 strain was investigated (or the nisin itself) on *Clostridium tyrobutyricum* using as a model system, a cheddar cheese matrix [12].
